# Occupational-circadian disruption and physical inactivity conjointly amplify fatty liver risk: based on liver ultrasound transient elastography

**DOI:** 10.13075/ijomeh.1896.02822

**Published:** 2026

**Authors:** Qian Zhang, Qing Qing Sun, Xuan Gu, Hao Cheng

**Affiliations:** 1 Xi'an Children's Hospital, Department of Ultrasound, Xi'an, China; 2 Lintong Rehabilitation and Recuperation Center, One Departments of Convalescence, Xi'an, China; 3 Xi'an Hospital of Civil Aviation, Infection Management and Control Office, Xi'an, China; 4 Shaanxi Cancer Hospital Affiliated to Xi'an Jiaotong University, Department of Ultrasonography, Xi'an, China

**Keywords:** CAP, physical activity, occupational health, fatty liver disease, shift work, work schedules

## Abstract

**Objectives:**

This study investigated the conjointly negative effects of floating shift work and physical inactivity on fatty liver disease (FLD) using ultrasound-based hepatic steatosis assessment.

**Material and Methods:**

Weighted multivariable logistic regression models were used to evaluate associations between work shifts, physical activity (PA), and FLD. Stratified and subgroup analyses were conducted to assess effect modification. Linear and quadratic regression models explored threshold effects of metabolic equivalent task (MET) levels on the degree of hepatic steatosis across shift types.

**Results:**

Data from 653 participants revealed that compared with the control group (controlled attenuation parameter – CAP <248), the case group (CAP ≥ 248) had a significantly higher prevalence of floating shifts (53.8% vs. 43.5%, p = 0.008) and physical inactivity (27.8% vs. 19.1%, p = 0.032). In multivariable-adjusted models, floating shifts independently increased FLD risk (odds ratio (OR) = 1.40, p = 0.019), while PA showed a borderline association with lower FLD risk (OR = 0.57, p = 0.056). Strikingly, compared to the reference group (physically active day-shift workers), physically inactive floating-shift workers had a higher risk of FLD (OR = 2.01, p = 0.049), highlighting the effects of occupational and behavioral factors. Subgroup analyses identified advanced age (OR = 2.42, p = 0.034), obesity (OR = 12.87, p < 0.001), hypertension (OR = 2.66, p = 0.028), and diabetes (OR = 6.21, p = 0.017) as critical risk factors among floating shift workers. At moderate METs (200–400 h/week), CAP values between floating and day shift workers converged with minimal differences in the quadratic regression model. Sensitivity analyses confirmed the robustness of the primary findings: the FLD risk persisted after excluding liver diseases and with an alternative PA definition (≥22 MET-h/week). A stricter CAP threshold attenuated the shift work association. Notably, adjusting for dietary pattern attenuated but did not eliminate the significant link between shift work and FLD (OR = 1.39, p = 0.030).

**Conclusions:**

These findings emphasize the urgent need for workplace interventions targeting shift schedules to mitigate FLD risk, particularly in high-risk occupational populations.

## Highlights

- Floating shifts raised fatty liver disease (FLD) risk 40% vs. day shifts (OR = 1.40, p = 0.019).
- Floating shifts plus inactivity doubled FLD risk (OR = 2.01, p = 0.049).
- Moderate activity (200–400 metabolic equivalent task hours per week) reduced CAP gap between shifts.

## INTRODUCTION

Emerging evidence underscores occupational circadian disruption as a critical determinant of metabolic dysregulation, with shift work increasingly recognized as a risk amplifier for cardiometabolic disorders. Over 20% of the global workforce engages in non-traditional schedules, particularly in healthcare, utilities, food services, manufacturing, and transportation sectors, where floating shifts (variable hours across mornings, days, and nights) predominate [1,2]. Such schedules disrupt circadian rhythms, impair hepatic lipid metabolism, and exacerbate development of fatty liver disease (FLD) [3].

Mechanistically, circadian disruption may compound metabolic risk through multiple pathways. At the systemic level, it can impair nervous system regulation, reducing daytime excitability, lowering motivation for physical activity (PA), and decreasing resting energy expenditure, thereby diminishing the benefits of exercise [4,5]. At the metabolic level, it is known to promote hepatic lipogenesis while reducing fatty acid β-oxidation and glucose metabolism [6,7]. Concurrently, physical inactivity-prevalent in >27% of shift workers due to fatigue and time constraints-independently promotes hepatic steatosis by creating an energy surplus and impairing mitochondrial function [8,9]. In contrast, regular PA helps maintain circadian alignment, with its timing acting as a secondary zeitgeber similar to light exposure [10]. Moreover, exercise can counteract these detrimental processes by enhancing insulin sensitivity, upregulating mitochondrial biogenesis, and improving lipid metabolism to reduce hepatic fat deposition [11–13]. Despite mechanistic plausibility, epidemiological evidence quantifying the combined associations of these occupational-behavioral factors remains sparse.

Current literature predominantly examines fixed night shifts, neglecting the distinct metabolic consequences of floating schedules characterized by irregular circadian realignment. Furthermore, existing studies often overlook the modifying role of PA, though experimental data suggest exercise may partially mitigate floating shift work-induced metabolic disturbances [14]. These limitations hinder the development of circadian-aligned interventions, particularly given the rising global burden of FLD, now affecting 25% of adults worldwide [15]. The authors study addresses these limitations through population-level analysis of floating shifts and PA using vibration-controlled transient elastography (VCTE) to assess hepatic steatosis.

Leveraging the 2017–2018 National Health and Nutrition Examination Survey (NHANES) cycle-the first nationally representative dataset incorporating detailed shift classifications – the hypothesizes are:

– floating shifts independently elevate FLD risk beyond traditional day shifts,
– physical inactivity may exacerbate floating shift work-related hepatic steatosis.

By elucidating these interfering factors, The authors' findings aim to inform precision workplace health strategies targeting high-risk occupational cohorts.

## MATERIAL AND METHODS

### Study design

**Figure 1.**
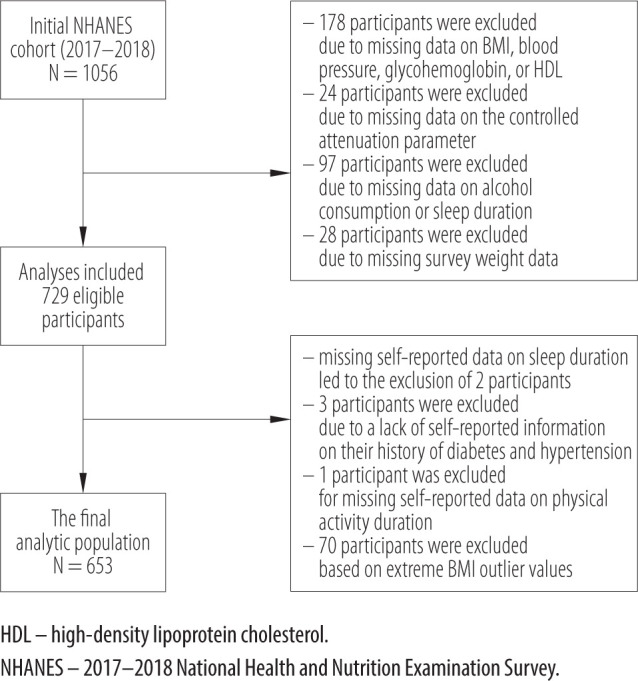
Flowchart of participant inclusion and exclusion in the study on negative effects of floating shift work and physical inactivity on fatty liver disease, 2017–2018, USA

This cross-sectional analysis utilized data from the 2017–2018 NHANES cycle. The protocols for each NHANES cycle were approved by the national center for health statistics of the centers for disease control and prevention institutional review board and all participants provided written informed consent. Use of NHANES data constitutes secondary data analysis and therefore exempts this protocol from Centers for Disease Control and Prevention (CDC) Institutional Review Boards (IRB) approval. The authors restricted analysis to individuals reporting either fixed dayshift or floating work schedules during the preceding three months (N = 1056). Participants with incomplete data on critical variables (including sleep duration, PA, work schedules, diabetes/hypertension status, BMI, alcohol consumption, systolic blood pressure [SY], glycohemoglobin, high-density lipoprotein cholesterol [HDL-C], controlled attenuation parameter [CAP], and weight [N = 333]) were excluded. Subsequent processing involved Z-score normalization of BMI, with exclusion of extreme outliers (absolute Z-score >1.5, N = 70). The final analytical sample included 653 participants. The specific inclusion and exclusion process is shown in Figure 1.

### Measurement of CAP

In the NHANES 2017–2018 survey, liver steatosis was assessed using VCTE with the FibroScan 502 V2 Touch system (Waltham, United States). The device, equipped with medium or extra-large probes selected through realtime guidance, measured CAP. Elevated CAP reflected increased hepatic fat content. In this study, participants with CAP ≥248 dB/m were defined as the case group with hepatic steatosis, and those with CAP <248 dB/m were defined as the control group without hepatic steatosis. The authors' analysis adopted 2 established CAP thresholds: 248 dB/m for routine steatosis detection and 285 dB/m for sensitivity evaluation [16].

### Definition of floating shift work and PA

Analyses were restricted to the 2017–2018 NHANES cycle due to exclusive availability of work schedule data. Participants self-reported their primary work schedules over the preceding 3 months, categorized as day shift (traditional 9 a.m. – 5 p.m.) or floating shift (variable hours across mornings, days, and nights).

Different types of exercise correspond to different MET values. In NHANES, MET values are assigned based on activity type. Physical activity was quantified as total MET-h/week, calculated as the sum of hours spent in each activity domain multiplied by its domain-specific MET value. For the authors' analysis, this involved 5 domains: vigorous work (8.0 METs), moderate work (4.0 METs), walking/cycling transportation (4.0 METs), vigorous leisure (8.0 METs), and moderate leisure (4.0 METs). Participants were classified as physically active if achieving ≥10 MET-h/week, aligning with WHO recommendations for adult PA thresholds [17].

### Covariates

Covariates were:

– sex (male/female);
– race (Hispanic/non-Hispanic);
– advanced age defined as >50 years;
– BMI (normal: <25 kg/m², overweight: 25–29.9 kg/m², obese: ≥30 kg/m²);
– sleep duration (6-8 h, <6 h or >8 h);
– alcohol consumption defined as any intake (≥1 occasion) vs. none within the past year;
– diabetes mellitus diagnosed by ≥1 of: self-reported diagnosis, hypoglycemic/insulin therapy, HbA_1c_ ≥6.5%, or fasting glucose ≥7.0 mmol/l;
– hypertension defined as ≥1 of: self-reported diagnosis, antihypertensive medication use, or blood pressure ≥140/90 mm Hg.

To account for overall dietary pattern, a composite unhealthy diet score was calculated for each participant using day one 24-hour dietary recall data. This score was derived as the sum of the standardized Z-scores of total calorie, saturated fat, and total sugar intake, and was included as a continuous covariate in relevant models to control for its potential confounding effect.

### Statistical analyses

All analyses accounted for the complex sampling design of NHANES by applying survey weights to ensure nationally representative estimates. Weighted multivariable logistic regression models were applied to evaluate associations between shift work patterns, PA and FLD. Stratified analyses restricted to floating-shift workers were performed to evaluate effect modification across all covariates. Threshold effects of MET levels on CAP values were explored using linear and quadratic regression models, stratified by shift type. Sensitivity analyses were performed to evaluate the robustness of associations:

– exclusion of participants with pre-existing liver pathologies to minimize confounding by baseline hepatic dysfunction;
– application of a stricter diagnostic threshold for hepatic steatosis (CAP ≥285 dB/m) to assess consistency across disease severity classifications;
– using an alternative, data-driven definition of physical inactivity to assess the robustness of the primary PA categorization (in this analysis, participants were dichotomized based on the sample median of weekly MET-h (<22 MET-h/week), rather than the predefined clinical cutoff (<10 MET-h/week) used in the main analysis);
– including the pre-calculated unhealthy diet score (see Covariates) as a covariate to examine whether the association between shift work and FLD is modified by overall diet quality.

To assess whether BMI, diabetes, and hypertension act as mediators rather than confounders, the authors conducted a Bayesian mediation analysis with 100 simulation iterations. P-value < 0.05 was considered statistically significant.

## RESULTS

### Population characteristics

Compared with controls, cases had a higher prevalence of physical inactivity (27.8% vs. 19.1%, p = 0.032). Significant disparities (p < 0.05) were noted in sex (male: 65.1% vs. 48.6%), age (advanced: 41.1% vs. 25.6%), race (Hispanic: 18.5% vs. 12.1%), BMI (obesity: 48.4% vs. 15.4%), drink (88.4% vs. 92.9%), hypertension (38.0% vs. 18.0%), diabetes (13.3% vs. 2.2%), and metabolic profiles (SY, diastolic blood pressure, HDL, glucose, glycohemoglobin). Sleep duration showed no difference (Table 1). In addition, cases had a higher prevalence of floating shifts (53.8% vs. 43.5%, p = 0.008)

**Table 1:** Weighted baseline characteristics stratified by hepatic steatosis status in the participants, 2017–2018, USA

Variable	Participants (N = 653)		p
	total	control (N = 318)	
Sociodemographic [n (%)]			
sex			0.007
male	378 (56.4)	157 (48.6)	
age			0.012
advanced	238 (32.9)	88 (25.6)	
race			0.005
Hispanic	160 (15.1)	58 (12.1)	
BMI [kg/m^2^]			<0.001
overweight	228 (33.6)	103 (30.4)	
obese	215 (30.9)	52 (15.4)	
Lifestyle factors [n (%)]			
drink			0.024
no	81 (9.2)	34 (7.1)	
MET level			0.032
inactive	176 (23.2)	75 (19.1)	
sleep duration			0.411
non-optimal (<6 or >8 h)	264 (37.1)	137 (39.6)	
Medical			
hypertension [n (%)]			0.001
yes	209 (27.4)	66 (18.0)	
diabetes [n (%)]			<0.001
yes	90 (7.4)	19 (2.2)	
blood pressure [mm Hg] (Me (Q1, Q3))			
systolic	117.33 (108.67, 126.78)	114.00 (106.95, 125.38)	0.001
diastolic	73.67 (66.67, 80.00)	71.00 (64.33, 76.67)	<0.001
HDL-cholesterol [mg/dl] (Me (Q1, Q3))	53.00 (44.00, 64.00)	58.00 (48.00, 69.00)	<0.001
glucose [mg/dl] (Me (Q1, Q3))	101.00 (96.00, 108.00)	99.00 (94.00, 104.00)	<0.001
glycohemoglobin [%] (Me (Q1, Q3))	5.40 (5.10, 5.60)	5.30 (5.10, 5.50)	<0.001

BMI – body mass index; MET – metabolic equivalent task.

Unweighted n, all other analyses are weighted.

### Association between floating shift and PA with FLD risk

In Table 2, after adjusting for age, gender, and race, floating shift workers had higher FLD risk compared with day shifts (model 2: odds ratio [OR] = 1.40, 95% confidence interval [CI]: 1.07–1.82, p = 0.019). Active PA reduced risk (model 2: OR = 0.57, 95% CI: 0.32–1.02, p = 0.056). Compared to active-day-shift workers, elevated risks were observed for inactive-day-shift (OR = 2.54, 95% CI: 1.04–6.18, p = 0.042), active-floating-shift (OR = 1.74, 95% CI: 1.16–2.60, p = 0.013), and inactive-floatingshift workers (OR = 2.01, 95% CI: 1.00–4.03, p = 0.049). After further adjustment for drink and sleep duration (model 3), a statistically significant association between floating shift work and FLD remained. Furthermore, compared to the active-day-shift workers, only the active-floating-shift group maintained a statistically significant association. In model 4, the associations were attenuated though trends persisted. Interaction tests revealed no significant interaction between floating shift work and PA (fully adjusted model: interaction OR = 0.95, 95% CI: 0.17–5.42, p = 0.930).

**Table 2:** Weighted associations of work shifts and physical activity (PA) with fatty liver disease, 2017–2018, USA

Indicator	Paticipants (N = 653)		Model 1			Model 2			Model 3			Model 4		
	control group (N = 318)	studied group (N = 335)	OR	95% CI	p	OR	95% CI	p	OR	95% CI	p	OR	95% CI	p
Shift														
day	178	159	ref.			ref.			ref.			ref.		
floating	140	176	1.51	1.13–2.02	0.009	1.40	1.07–1.82	0.019	1.42	1.03–1.96	0.036	1.31	0.76–2.24	0.260
PA														
inactive	75	101	ref.			ref.			ref.			ref.		
active	243	234	0.61	0.39–0.96	0.034	0.57	0.32–1.02	0.056	0.57	0.31–1.03	0.061	0.75	0.36–1.55	0.350
Shift and PA														
day shift and														
active PA	136	98	ref.			ref.			ref.			ref.		
inactive PA	42	61	2.39	1.18–4.86	0.020	2.54	1.04–6.18	0.042	2.59	0.99–6.78	0.052	1.35	0.35–5.23	0.537
floating shifts and														
active PA	107	136	1.87	1.22–2.87	0.008	1.74	1.16–2.60	0.013	1.78	1.10–2.88	0.025	1.32	0.55–3.19	0.392
inactive PA	33	40	2.09	1.09–4.01	0.030	2.01	1.00–4.03	0.049	2.06	0.98–4.32	0.054	1.87	0.54–6.50	0.208
PA × shift (interaction)	318	335	2.14	0.72–6.42	0.156	2.19	0.68–7.04	0.162	2.24	0.67–7.47	0.158	0.95	0.17–5.42	0.930

Model 1 – not adjusted; model 2 – adjusted by age, gender, race; model 3 – adjusted by age, gender, race, drink, sleep duration; model 4 – adjusted by age, gender, race, BMI, drink, sleep duration, hypertension, diabetes.

Mediation analysis was conducted to assess whether BMI, diabetes, and hypertension mediate the association between shift work and FLD. For diabetes and hypertension, the direct and total effects were significant, but the indirect effects were not. For BMI, no significant total, direct, or indirect effects were observed (Table 3).

**Table 3:** Bayesian mediation analysis of body mass index (BMI), diabetes, and hypertension on the association between shift work and fatty liver, 2017–2018, USA

Variable and effect type	Estimate (95% CI)	p
BMI		
total	0.08 (0.00–0.15)	0.060
direct	0.06 (–0.00–0.13)	0.060
indirect	0.02 (–0.01–0.06)	0.220
Hypertension		
total	0.09 (0.01–0.15)	0.020
direct	0.09 (0.01–0.15)	0.020
indirect	0.00 (–0.02–0.02)	0.860
Diabetes		
total	0.09 (0.01–0.15)	0.020
direct	0.09 (0.01–0.15)	0.020
indirect	0.00 (–0.02–0.02)	0.920

### Subgroup analyses

In weighted analyses of floating shift workers, significant risk factors for FLD included age ≥51 years (OR = 2.42, p = 0.034), overweight (OR = 5.78, p = 0.006), obesity (OR = 12.87, p < 0.001), hypertension (OR = 2.66, p = 0.028), and diabetes (OR = 6.21, p = 0.017). Females (OR = 0.57, p = 0.052) and drinkers (OR = 0.46, p = 0.059) showed marginally reduced risks. Other variables (race, MET level, sleep duration) exhibited no significant associations (Table 4).

**Table 4:** Weighted subgroup analyses of risk factors for fatty liver disease among floating shift workers, 2017–2018, USA

Variable	Paticipants (N = 316)		OR (95% CI)	p
	control group (N = 140)	studied group (N = 176)		
Sociodemographic				
sex				
male	66	125	ref.	
female	74	51	0.57 (0.33–1.01)	0.052
age				
20–50 years	100	96	ref.	
51–80 years	40	80	2.42 (1.08–5.42)	0.034
race				
Hispanic	31	47	ref.	
non-Hispanic	109	129	0.95 (0.61–1.47)	0.804
body mass index				
normal	67	24	ref.	
overweight	48	65	5.78 (1.84–18.17)	0.006
obese	25	87	12.87 (4.27–38.8)	<0.001
Lifestyle factors				
drink				
no	12	27	ref.	
yes	128	149	0.46 (0.2–1.04)	0.059
physical activity				
inactive	33	40	ref.	
active	107	136	0.9 (0.43–1.89)	0.759
sleep duration				
<6 h or >8 h	59	76	ref.	
6–8 h	81	100	1.35 (0.64–2.85)	0.399
Medical				
hypertension				
no	110	103	ref.	
yes	30	73	2.66 (1.13–6.25)	0.028
diabetes				
no	131	141	ref.	
yes	9	35	6.21 (1.46–26.43)	0.017

### Association between continuous MET levels and median CAP

**Figure 2.**
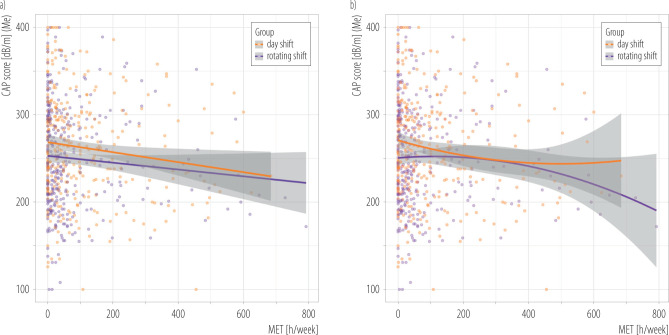
Association between metabolic equivalent task (MET) level and median (Me) controlled attenuation parameter (CAP) stratified by work shifts: a) linear regression model, b) quadratic regression model, 2017–2018, USA

The association between weekly MET level and CAP values exhibited distinct patterns between floating shift and day shift workers. In the linear regression model, floating shift workers demonstrated a steeper negative slope compared to day shift workers, with CAP levels consistently higher in floating shifts across most METs ranges. In the quadratic regression model, at moderate METs levels (200–400 h/week), CAP values between floating and day shift workers converged, showing minimal differences. However, beyond this range (METs <200 h/week or >400 h/week), floating shift workers displayed significantly elevated CAP values compared to day shifts, suggesting a threshold effect of PA on hepatic steatosis mitigation in this occupational subgroup (Figure 2).

### Sensitivity analysis

After excluding individuals with pre-existing liver diseases (N = 56), weighted analyses (Table 5) demonstrated similar trends to Table 2: floating shifts remained associated with elevated FLD risk (model 1: OR = 1.43, 95% CI: 1.08–1.90, p = 0.016), though association strength attenuated compared to Table 2. Physical activity retained a favorable trend (model 1: OR = 0.61, p = 0.030). Stratified analyses revealed consistent risk elevations for inactive-day-shift (model 1: OR = 2.28, p = 0.027), active-float ing-shift (model 1: OR = 1.75, p = 0.015), and inactive-floating-shift workers (model 1: OR = 2.03, p = 0.040), mirroring Table 2. Full adjustment (model 3) further diluted associations. The results reinforce the robustness of floating shift work and inactivity effects on FLD, even after excluding baseline liver pathologies. Table 5 also evaluates associations between floating shif work, PA, and FLD using a stricter CAP threshold (≥285 dB/m). Similar to Table 2 (CAP ≥248 dB/m), floating shift workers showed elevated FLD risk compared to day-shift workers (model 1: OR = 1.34, 95% CI: 0.83–2.15), but this association attenuated to non-significance. Active PA remained favorable in both tables, though its effect weakened in Table 5 (model 2: OR = 0.40 vs. 0.57 in Table 2). Stratified analyses mirrored Table 2: inactive-day-shift workers (OR = 4.32, p = 0.004) and active-floating-shift workers (OR = 1.86, p = 0.015) faced higher FLD risks versus active-day-shift workers. However, inactive-floating-shift workers exhibited no significance in Table 5 (OR = 2.64, p = 0.100), diverging from Table 2's clear risk elevation (OR = 2.01, p = 0.049). Compared to Table 2, Table 5 employed a stricter diagnostic threshold, excluding mild steatosis cases.

**Table 5:** Weighted associations of work shifts and physical activity (PA) with fatty liver disease in sensitivity analyses: excluding pre-existing liver conditions or using a stricter threshold (CAP ≥285 dB/m), 2017–2018, USA

Indicator	Paticipants (N = 653)		Model 1			Model 2			Model 3		
	control group (N = 318)	studied group (N = 335)	OR	95% CI	p	OR	95% CI	p	OR	95% CI	p
After excluding pre-existing liver conditions (N = 597)											
shift											
day	164	151	ref			ref			ref		
floating	126	156	1.43	1.08–1.9	0.016^*^	1.31	1.00–1.73	0.053	1.25	0.72–2.17	0.341
PA											
inactive	72	96	ref			ref			ref		
active	218	211	0.61	0.39–0.95	0.030^*^	0.58	0.33–1.01	0.053	0.73	0.35–1.54	0.333
day shift and active PA	123	92	ref			ref			ref		
day shift and inactive PA	41	59	2.28	1.12–4.65	0.027^*^	2.37	0.99–5.67	0.052	1.29	0.33–5.01	0.597
floating shifts and active PA	95	119	1.75	1.14–2.68	0.015^*^	1.59	1.05–2.41	0.031^*^	1.23	0.49–3.04	0.528
floating shifts and inactive PA	31	37	2.03	1.04–3.96	0.040^*^	1.93	0.95–3.91	0.064	1.9	0.52–6.92	0.213
Using a stricter threshold (CAP ≥285 dB/m)											
shift											
day	233	104	ref			ref			ref		
floating	198	118	1.34	0.83–2.15	0.212	1.26	0.78–2.05	0.307	1.16	0.57–2.34	0.614
PA											
inactive	104	72	ref			ref			ref		
active	327	150	0.43	0.24–0.77	0.007	0.4	0.20–0.79	0.013	0.48	0.18–1.25	0.106
day shift and active PA	175	59	ref			ref			ref		
day shift and inactive PA	58	45	3.99	1.92–8.31	0.001	4.32	1.82–10.25	0.004	2.48	0.55–11.12	0.15
floating shifts and active PA	152	91	1.96	1.24–3.09	0.007	1.86	1.16–2.97	0.015	1.41	0.53–3.77	0.346
floating shifts and inactive PA	46	27	2.64	0.86–8.12	0.085	2.64	0.80–8.78	0.1	2.66	0.29–24.59	0.256

Model descriptions are provided in Table 2.

* p < 0.05.

To examine the association between MET and FLD across different activity levels, the authors classified participants based on the median MET-h/week (≥22 defined as the active group) and also performed log-transformation of MET values. Compared with the inactive group, regular PA was significantly associated with a reduced risk of fatty liver after adjusting for age, sex, and ethnicity. However, no statistically significant association was observed in the unadjusted model or in the fully adjusted model. Similarly, log-transformed MET was not significantly associated with the outcome (Table 6).

**Table 6:** Weighted associations of physical activity (PA) and fatty liver disease based on different physical activity cutoffs, 2017–2018, USA

Model	OR	95% CI	p
Model 1			
log (metabolic equivalents)	0.95	0.89–1.02	0.150
inactive PA	ref.		
active PA	0.76	0.56–1.03	0.077
Model 2			
log (metabolic equivalents)	0.94	0.86–1.02	0.127
inactive PA	ref.		
active PA	0.70	0.52–0.93	0.020
Model 3			
log (metabolic equivalents)	0.98	0.88–1.10	0.678
inactive PA	ref.		
active PA	0.97	0.57–1.65	0.899

Model descriptions are provided in Table 2.

Physical activity was categorized based on the median value of MET-h/week.

The inactive PA group was defined as having MET-h/week <22, and the active PA group – MET-h/week ≥22.

This sensitivity analysis investigated the association between floating shift work and FLD after adjusting for the overall diet pattern. In the total population, where the unhealthy diet score was included as a continuous covariate, floating shift work showed a significant association in the basic model (model 1: OR = 1.39, 95% CI: 1.04–1.86). This association was attenuated and became non-significant in the fully adjusted model (model 3: OR = 1.28, 95% CI: 0.72–2.28). In the subgroup analysis stratified by the median diet score, a positive association was observed among participants with a favorable diet in models 1 and 2, but not in their fully adjusted model 3 (OR = 1.82, 95% CI: 0.94–3.55, p = 0.068). No significant association was found in the unfavorable diet group. The p-value for interaction was 0.091 (Table 7).

**Table 7:** Weighted association of shift work with fatty liver, overall and stratified by unhealthy diet score, 2017–2018, USA

Analysis dimension	OR	95% CI	p
Total population^a^			
model 1	1.39	1.04–1.86	0.030
model 2	1.32	0.97–1.80	0.074
model 3	1.28	0.72–2.28	0.298
Diet group			
favorable (score ≤–0.43)			
model 1	2.48	1.39–4.44	0.005
model 2	2.56	1.48–4.43	0.003
model 3	1.82	0.94–3.55	0.068
unfavorable (score >–0.43)			
model 1	0.90	0.43–1.86	0.749
model 2	0.86	0.39–1.87	0.675
model 3	1.02	0.37–2.81	0.961
Shift × diet group			0.091

Model descriptions are provided in Table 2.

Subgroup analysis – participants were dichotomized by the median diet score (–0.43).

Models within each subgroup did not include the diet score.

The p for interaction was from a multiplicative term (shift work × diet score group) in unadjusted model.

Analyses were restricted to 639 participants with complete data on all variables and reliable dietary recall. Participants with a 0 dietary day-1 sample weight were excluded.

a The unhealthy diet score (continuous) was included as a covariate in all models.

## DISCUSSION

The authors' nationally representative analysis reveals novel insights into the occupational epidemiology of FLD. Floating shift workers exhibited 40% greater FLD risk than day shift workers, consistent with murine models showing that erratic light cycles promote hepatic lipid accumulation and shift the circadian rhythmicity of hepatic acetyl-CoA carboxylase mRNA expression [18]. The observed risk escalation in physically inactive floating shift workers suggests PA mitigates but does not fully counteract circadian disruption effects. Although previous murine studies have proposed mechanisms such as exercise-induced mitochondrial enhancement, mitochondrial unfolded protein response (UPRmt) reversal, and fibroblast growth factor 21 (FGF21) secretion [19], these biological pathways were not directly measured in the authors' human epidemiological study. Notably, the observed favorable window at 200–400 MET-h/week, substantially higher than the current WHO guideline of ≥150 MET-h/week, requires cautious interpretation. First, the authors' MET-h estimate aggregated both leisure-time and occupational PA. Second, this range represents a statistical inflection point from the authors' threshold analysis rather than a minimum recommended dose. Therefore, while the authors' findings suggest a dose-response relationship, they do not advocate that all floating shift workers must achieve 200–400 MET-h/week. The optimal dose should be refined in future large-scale prospective studies.

The pronounced association between floating shift work and elevated FLD risk (OR = 1.40, p = 0.019) underscores the metabolic consequences of irregular circadian rhythms. Weight gain might not be merely a confounder but could also serve as a mediator in the causal pathway between circadian disruption and hepatic steatosis. The biological plausibility of this mediation is supported by converging mechanisms:

– circadian misalignment directly promotes metabolic dysregulation, including impaired glucose metabolism and increased systemic inflammation [20],
– these alterations, coupled with melatonin deficiency and resultant oxidative stress, create an endocrine and metabolic milieu conducive to weight gain and ectopic fat deposition [21–23].

A prospective analysis of 281–280 participants demonstrated that shift work, particularly permanent night shifts, was associated with a 27% higher risk of incident nonalcoholic fatty liver disease (NAFLD) (95% CI: 1.08–1.48). This association followed a dose-response pattern and was not modified by genetic susceptibility, supporting that floating shift work may contribute to hepatic steatosis development [24]. However, caution should be exercised in interpreting the results of the mediation analysis presented in this article. The cross-sectional design of this study does not allow to determine the time sequence between exposure (floating shift work), media (BMI, diabetes, hypertension) and outcomes (FLD). Therefore, the observed direct and indirect effects may be influenced by reverse causality or unmeasured confounding factors. Future prospective studies are necessary to confirm the potential mediating role of these metabolic factors.

While physical inactivity was associated with higher FLD risk across all shift types (OR = 2.01–2.54, p < 0.05), the authors' threshold analysis suggested a potential favorable window at 200–400 MET-h/week, where CAP values converged between floating and day shift workers. This convergence suggests that sufficient PA may mitigate the specific hepatic risk imposed by circadian misalignment. The underlying mechanisms are multifaceted but were not directly assessed in this study. Based on existing literature, plausible pathways include increased energy expenditure and hepatic fatty acid oxidation [25], as well as potential modulation of circadian signaling via adenosine monophosphate-activated protein kinase (AMPK) phosphorylation of clock proteins [26,27]. Exercise may also suppress inflammatory pathways [e.g., myeloid differentiation protein 2-toll-like receptor 4 (MD2-TLR4)/nuclear factor kappa-B (NF-κB)] and improve hepatic insulin sensitivity [28–30]. However, these molecular and cellular processes remain speculative in the context of the authors' observational data, and direct measurements (e.g., circadian gene expression, inflammatory markers, or insulin sensitivity indices) are needed to confirm mediation. Thus, the authors' epidemiological findings are supported by a plausible physiological framework: floating shift work disrupts circadian-metabolic coupling to promote FLD, while adequate PA can counteract these effects by restoring circadian signaling, reducing inflammation, and improving hepatic insulin action. The independent protective role of PA should be interpreted with caution.

Subgroup analyses revealed that night shift workers with metabolic risk factors (age >50 years, obesity, hypertension, diabetes) had an increased risk of FLD. Both obesity, hypertension, diabetes and floating shift work are established risk factors for metabolic syndrome (MetS). They may be associated with an increased risk of FLD progression through multifactorial mechanisms, including aggravated insulin resistance and chronic lowgrade inflam mation [31]. Prolonged night-shift tenure in aging workers induces chronic circadian disruption that dysregulates hepatometabolic processes. The pathophysiological nexus between obesity and FLD has been extensively validated through multidimensional investigations encompassing extensive studies [32,33]. Epidemiological studies have established hypertension as an independent predictor of NAFLD. Notably, prehypertension also demonstrated an association with NAFLD (with elevated ORs observed across prehypertensive blood pressure ranges) [34]. In diabetic floating shift workers, sleep deprivation and erratic eating patterns may worsen insulin resistance, promoting fatty liver by upregulating sterol regulatory element-binding protein-1c-driven lipogenesis [35]. The authors' stratified analyses confirm prior findings, and these comorbidities represent a critical consideration for workplace health screenings.

In response to the important consideration that overall diet quality could confound the observed association, the authors conducted additional analyses using detailed dietary recall data. When the authors adjusted for a composite “unhealthy diet score,” the association between floating shift work and FLD was attenuated in the full cohort. This suggests that concurrent dietary patterns account for some of the apparent risk linked to floating shift work. Interestingly, when the authors stratified participants by this diet score, the association was significant only in the subgroup with a favorable diet, and was absent in those with an unfavorable diet. This pattern hints that the negative impact of circadian disruption on the liver might be most evident when baseline metabolism is relatively healthy. One possible explanation is that a liver maintained by a better diet has greater metabolic reserve to cope with stressors; when floating shift work disrupts circadian rhythms, this reserve is challenged, making the effect detectable. Conversely, a consistently poor diet may itself impose a chronic metabolic burden on the liver, leaving little additional room for floating shift work to exert a separate, measurable effect.

Several limitations must be acknowledged. First, the reduction in sample size due to missing data may introduce bias. Second, floating shift work and PA were self-reported and subject to recall bias. Thirdly, circadian disruption was measured only as floating shift work over three months, without capturing its duration, frequency, or sleep quality. Fourth, other unmeasured factors, including psychological stress, social isolation, and irregular dietary patterns with potential gut microbiota alterations, may have further modified the observed associations, but were not assessed in this study.

## CONCLUSIONS

This population-based study suggests an association that floating shift work is associated with increased risk of hepatic steatosis, with effect magnitudes surpassing those of traditional metabolic drivers. While the cross-sectional design revealed robust associations, the causal direction requires validation through longitudinal cohort studies.

## References

[1] Hanprathet N, Lertmaharit S, Lohsoonthorn V, Rattananupong T, Ammaranond P, Jiamjarasrangsi W. Increased risk of type 2 diabetes and abnormal FPG due to shift work differs according to Gender: a retrospective cohort study among Thai workers in Bangkok, Thailand. Diabetes Metab Syndr Obes. 2019; 12:2341–54. 10.2147/dmso.S219524. 32009809 PMC6859211

[2] Wang Y, Li R, Ye Q, Fei D, Zhang X, Huang J, et al. Circadian disruption by simulated shift work aggravates periodontitis via orchestrating BMAL1 and GSDMD-mediated pyroptosis. Int J Oral Sci. 2025;17(1):14. 10.1038/s41368-024-00331-x. 40000642 PMC11861291

[3] Tahara Y, Shibata S. Circadian rhythms of liver physiology and disease: experimental and clinical evidence. Nat Rev Gastroenterol Hepatol. 2016;13(4):217–26. 10.1038/nrgastro.2016.8. 26907879

[4] Zhang C, Zeng P, Tan J, Sun S, Zhao M, Cui J, et al. Relationship of problematic smartphone use, sleep quality, and daytime fatigue among quarantined medical students during the COVID-19 Pandemic. Front Psychiatry. 2021;12:755059. 10.3389/fpsyt.2021.755059. 34858229 PMC8631394

[5] Harmsen JF, Wefers J, Doligkeit D, Schlangen L, Dautzenberg B, Rense P, et al. The influence of bright and dim light on substrate metabolism, energy expenditure and thermoregulation in insulin-resistant individuals depends on time of day. Diabetologia. 2022;65(4):721–32. 10.1007/s00125-021-05643-9. 35106618 PMC8894310

[6] Jiang X, Zheng J, Zhang S, Wang B, Wu C, Guo X. Advances in the involvement of gut microbiota in pathophysiology of NAFLD. Front Med (Lausanne). 2020;7:361. 10.3389/fmed.2020.00361. 32850884 PMC7403443

[7] Wang X, Rao J, Zhang L, Liu X, Zhang Y. Identification of circadian rhythm-related gene classification patterns and immune infiltration analysis in heart failure based on machine learning. Heliyon. 2024;10(6):e27049. 10.1016/j.heliyon.2024.e27049. 38509983 PMC10950509

[8] Fekedulegn D, Long DL, Service S, Gu JK, Innes KE. Shiftwork and leisure-time physical inactivity (LTPI) among U.S. workers. Chronobiol Int. 2025;42(1):1–13. 10.1080/07420528.2024.2437427. 39690873 PMC11835517

[9] He P, Deng Y, Dong S, Li H, Liu C, Ma Y, et al. Association of different domains of physical activity with diabetic kidney disease: a population-based study. Front Endocrinol (Lausanne). 2024;15:1364028. 10.3389/fendo.2024.1364028. 38863925 PMC11165133

[10] Shen B, Ma C, Wu G, Liu H, Chen L, Yang G. Effects of exercise on circadian rhythms in humans. Front Pharmacol. 2023; 14: 1282357. 10.3389/fphar.2023.1282357. 37886134 PMC10598774

[11] Cao J, Lei S, Zhao T, Xie Y, Zhou Z, Cheng S, et al. Changes in fat oxidation and body composition after combined exercise intervention in sedentary obese Chinese adults. J Clin Med. 2022;11(4). 10.3390/jcm11041086. PMC8879656 35207356

[12] Li N, Zhang L, Guo Q, Shi H, Gan Y, Wang W, et al. Aerobic exercise improves inflammation and insulin resistance in skeletal muscle by regulating miR-221-3p via JAK/STAT signaling pathway. Front Physiol. 2025;16:1534911. 10.3389/fphys.2025.1534911. 40070461 PMC11893602

[13] Rajewski P, Cieściński J, Rajewski P, Suwała S, Rajewska A, Potasz M. Dietary interventions and physical activity as crucial factors in the prevention and treatment of metabolic dysfunction-associated steatotic liver disease. Biomedicines. 2025;13(1). 10.3390/biomedicines13010217. PMC11760440 39857800

[14] Atkinson G, Fullick S, Grindey C, Maclaren D. Exercise, energy balance and the shift worker. Sports Med. 2008; 38(8): 671–85. 10.2165/00007256-200838080-00005. 18620467 PMC2784228

[15] Keles U, Ow JR, Kuentzel KB, Zhao LN, Kaldis P. Liver-derived metabolites as signaling molecules in fatty liver disease. Cell Mol Life Sci. 2022;80(1):4. 10.1007/s00018-022-04658-8. 36477411 PMC9729146

[16] Tian T, Zhang J, Xie W, Ni Y, Fang X, Liu M, et al. Dietary quality and relationships with metabolic dysfunction-associated fatty liver disease (MAFLD) among United States adults, results from NHANES 2017–2018. Nutrients. 2022; 14(21). 10.3390/nu14214505. PMC9659246 36364767

[17] Hu P, Zheng M, Huang J, Fan HY, Fan CJ, Ruan HH, et al. Effect of healthy lifestyle index and lifestyle patterns on the risk of mortality: A community-based cohort study. Front Med (Lausanne). 2022;9:920760. 10.3389/fmed.2022.920760. 36111119 PMC9468322

[18] Christie S, Vincent AD, Li H, Frisby CL, Kentish SJ, O'Rielly R, et al. A rotating light cycle promotes weight gain and hepatic lipid storage in mice. Am J Physiol Gastrointest Liver Physiol. 2018;315(6):G932–42. 10.1152/ajpgi.00020.2018. 30188750

[19] Yuan X, Sun W, Xu Y, Xiang M, Gao Y, Feng W, et al. Altered mitochondrial unfolded protein response and FGF21 secretion in MASLD progression and the effect of exercise intervention. Sci Rep. 2025;15(1):3686. 10.1038/s41598-025-87190-6. 39881157 PMC11779893

[20] Blankenship JM, Vetter C, Broussard JL. Impairments in glycemic control during Eastbound transatlantic travel in healthy adults. Sleep Adv. 2022;3(1):zpac009. 10.1093/sleepadvances/zpac009. 35601081 PMC9112920

[21] Dong J, Li M, Peng R, Zhang Y, Qiao Z, Sun N. ACACA reduces lipid accumulation through dual regulation of lipid metabolism and mitochondrial function via AMPKPPARα-CPT1A axis. J Transl Med. 2024;22(1):196. 10.1186/s12967-024-04942-0. 38395901 PMC10885411

[22] Zhou Y, Zhang C, Zhou Z, Zhang C, Wang J. Identification of key genes and pathways associated with PIEZO1 in bone-related disease based on bioinformatics. Int J Mol Sci. 2022; 23(9). 10.3390/ijms23095250. PMC9104149 35563641

[23] Gao T, Wang Z, Cao J, Dong Y, Chen Y. Melatonin ameliorates corticosterone-mediated oxidative stress-induced colitis in sleep-deprived mice involving gut microbiota. Oxid Med Cell Longev. 2021;2021:9981480. 10.1155/2021/9981480. 34257825 PMC8246302

[24] Huang H, Liu Z, Xie J, Xu C. Association between night shift work and NAFLD: a prospective analysis of 281,280 UK Biobank participants. BMC Public Health. 2023;23(1):1282. 10.1186/s12889-023-16204-7. 37400787 PMC10318710

[25] Chun SK, Lee S, Yang MJ, Leeuwenburgh C, Kim JS. Exercise-induced autophagy in fatty liver disease. Exerc Sport Sci Rev. 2017;45(3):181–6. 10.1249/jes.0000000000000116. 28419000 PMC5479347

[26] Ducloux D, Courivaud C. Prevention of post-transplant diabetes mellitus: towards a personalized approach. J Pers Med. 2022;12(1). 10.3390/jpm12010116. PMC8778007 35055431

[27] Lamia KA, Sachdeva UM, DiTacchio L, Williams EC, Alvarez JG, Egan DF, et al. AMPK regulates the circadian clock by cryptochrome phosphorylation and degradation. Science. 2009;326(5951):437–40. 10.1126/science.1172156. 19833968 PMC2819106

[28] Zhu W, Sahar NE, Javaid HMA, Pak ES, Liang G, Wang Y, et al. Exercise-induced irisin decreases inflammation and improves NAFLD by competitive binding with MD2. Cells. 2021; 10(12). 10.3390/cells10123306. PMC8699279 34943814

[29] da Cruz Rodrigues KC, Martins Pereira R, Peruca GF, Torres Barbosa LW, Ramos Sant'Ana M, Rosetto Muñoz V, et al. Short-term strength exercise reduces hepatic insulin resistance in obese mice by reducing PTP1B content, regardless of changes in body weight. Int J Mol Sci. 2021;22(12). 10.3390/ijms22126402. PMC8232771 34203825

[30] Malin SK, del Rincon JP, Huang H, Kirwan JP. Exercise-induced lowering of fetuin-A may increase hepatic insulin sensitivity. Med Sci Sports Exerc. 2014;46(11):2085–90. 10.1249/mss.0000000000000338. 24637346 PMC4640446

[31] Xie M, Tang H, Li F, Wu S, Dong Y, Yang Y, et al. Mediating roles of hsCRP, TNF-α and adiponectin on the associations between body fat and fatty liver disease among overweight and obese adults. Biology (Basel). 2021;10(9). 10.3390/biology10090895. PMC8469229 34571772

[32] Sahin K, Orhan C, Akdemir F, Tuzcu M, Sahin N, Yilmaz I, et al. Mesozeaxanthin protects the liver and reduces cardio-metabolic risk factors in an insulin resistant rodent model. Food Nutr Res. 2017;61(1):1353360. 10.1080/16546628.2017.1353360. 28804442 PMC5533124

[33] Lanthier N, Rodriguez J, Nachit M, Hiel S, Trefois P, Neyrinck AM, et al. Microbiota analysis and transient elastography reveal new extra-hepatic components of liver steatosis and fibrosis in obese patients. Sci Rep. 2021;11(1):659. 10.1038/s41598-020-79718-9. 33436764 PMC7804131

[34] Ma C, Yan K, Wang Z, Zhang Q, Gao L, Xu T, et al. The association between hypertension and nonalcoholic fatty liver disease (NAFLD): literature evidence and systems biology analysis. Bioengineered. 2021;12(1):2187–202. 10.1080/21655979.2021.1933302. 34096467 PMC8806441

[35] Samuel VT, Shulman GI. Nonalcoholic Fatty Liver Disease as a Nexus of Metabolic and Hepatic Diseases. Cell Metab. 2018; 27(1):22–41. 10.1016/j.cmet.2017.08.002. 28867301 PMC5762395

